# Epigenetic role of LINE-1 methylation and key genes in pregnancy maintenance

**DOI:** 10.1038/s41598-024-53737-2

**Published:** 2024-02-08

**Authors:** Veronica Tisato, Juliana A. Silva, Fabio Scarpellini, Roberta Capucci, Roberto Marci, Ines Gallo, Francesca Salvatori, Elisabetta D’Aversa, Paola Secchiero, Maria L. Serino, Giorgio Zauli, Ajay V. Singh, Donato Gemmati

**Affiliations:** 1 Department of Translational Medicine, University of Ferrara, 44121 Ferrara, Italy; 2 University Strategic Centre for Studies On Gender Medicine, University of Ferrara, 44121 Ferrara, Italy; 3 Centre Haemostasis & Thrombosis, University of Ferrara, 44121 Ferrara, Italy; 4 LTTA Centre, University of Ferrara, 44121 Ferrara, Italy; 5 Centre for Reproductive Medicine, CERM Hungaria, 00193 Rome, Italy; 6 Department of Medical Sciences, University of Ferrara, 44121 Ferrara, Italy; 7 Department of Environmental Sciences and Prevention, University of Ferrara, 44121 Ferrara, Italy; 8 Department of Chemical and Product Safety, German Federal Institute for Risk Assessment (BfR), 10589 Berlin, Germany

**Keywords:** Epigenomics, Epigenetics, Epidrugs, LINE-1 methylation, Retrotransposons, Inflammation, Cytokines, Spontaneous miscarriage, Pregnancy maintenance, Molecular medicine, Infertility, Genetics, Epigenetics, Risk factors

## Abstract

Spontaneous abortion is a pregnancy complication characterized by complex and multifactorial etiology. About 5% of childbearing women are globally affected by early pregnancy loss (EPL) and most of them experience recurrence (RPL). Epigenetic mechanisms and controlled inflammation are crucial for pregnancy maintenance and genetic predispositions may increase the risk affecting the maternal–fetal crosstalk. Combined analyses of global methylation, inflammation and inherited predispositions may contribute to define pregnancy loss etiopathogenesis. LINE-1 epigenetic regulation plays crucial roles during embryo implantation, and its hypomethylation has been associated with senescence and several complex diseases. By analysing a group of 230 women who have gone through pregnancy interruption and comparing those experiencing spontaneous EPL (n = 123; RPL, 54.5%) with a group of normal pregnant who underwent to voluntary interruption (VPI, n = 107), the single statistical analysis revealed significant lower (*P* < 0.00001) LINE-1 methylation and higher (*P* < 0.0001) mean cytokine levels (CKs: IL6, IL10, IL17A, IL23) in EPL. Genotyping of the following SNPs accounted for different EPL/RPL risk odds ratio: *F13A1* rs5985 (OR = 0.24; 0.06–0.90); *F13B* rs6003 (OR = 0.23; 0.047–1.1); *FGA* rs6050 (OR = 0.58; 0.33–1.0); *CRP* rs2808635/rs876538 (OR = 0.15; 0.014–0.81); *ABO* rs657152 (OR = 0.48; 0.22–1.08); *TP53* rs1042522 (OR = 0.54; 0.32–0.92); *MTHFR* rs1801133/rs1801131 (OR = 2.03; 1.2–3.47) and *FGB* rs1800790 (OR = 1.97; 1.01–3.87), although Bonferroni correction did not reach significant outputs. Principal Component Analysis (PCA) and logistic regression disclosed further SNPs positive/negative associations (e.g. *APOE* rs7412/rs429358; *FGB* rs1800790; *CFH* rs1061170) differently arranged and sorted in four significant PCs: PC1 (*F13A,* methylation, CKs); PC3 (*CRP*, *MTHFR,* age, methylation); PC4 (*F13B, FGA, FGB, APOE, TP53*, age, methylation); PC6 (*F13A, CFH, ABO, MTHFR, TP53*, age), yielding further statistical power to the association models. In detail, positive EPL risk association was with PC1 (OR = 1.81; 1.33–2.45; *P* < 0.0001) and negative associations with PC3 (OR = 0.489; 0.37–0.66; P < 0.0001); PC4 (OR = 0.72; 0.55–0.94; *P* = 0.018) and PC6 (OR = 0.61; 0.46–0.81; *P* = 0.001). Moreover, significant inverse associations were detected between methylation and CKs levels in the whole group (*r*_IL10_ = − 0.22; *r*_IL17A_ = − 0.25; *r*_IL23_ = − 0.19; *r*_IL6_ = − 0.22), and methylation with age in the whole group, EPL and RPL subgroups (*r*^*2*^_TOT_ = 0.147; *r*^*2*^_EPL_ = 0.136; *r*^*2*^_RPL_ = 0.248), while VPI controls lost significance (*r*^*2*^_VPI_ = 0.011). This study provides a valuable multilayer approach for investigating epigenetic abnormalities in pregnancy loss suggesting genetic-driven dysregulations and anomalous epigenetic mechanisms potentially mediated by LINE-1 hypomethylation. Women with unexplained EPL might benefit of such investigations, providing new insights for predicting the pregnancy outcome and for treating at risk women with novel targeted epidrugs.

## Introduction

Pregnancy loss represents the most common adverse event occurring during the first stages of implantation and recent epidemiological data reveal that the pooled risk of miscarriage is about 15% of all recognized pregnancies^1^. Early pregnancy loss (EPL) refers to spontaneous pregnancy termination within 12 weeks of gestational age, and several risk factors have been associated to recurrence (RPL) related to either maternal and/or fetal conditions^2^. Overall, we are referring to a complex process in which several inherited or acquired factors play a role including abnormal chromosome structure as aneuploidies, endocrine and immune dysregulation, lupus, reproductive features, prothrombotic state, together with other conditions such as age, ethnicity, previous miscarriages, environment and lifestyle^1–6^. Given that variability, knowledge regarding the exact causes and pathophysiological mechanisms involved in pregnancy maintenance as well as in the dynamic of the maternal–fetal interface have not been completely defined.

The uterine environment plays a crucial role in determining the pregnancy outcome since a proper blastocyst grafting in a receptive uterine endometrium represents the first step for successful embryo implantation also after in vitro fertilization (IVF)^7–9^. Therefore, pregnancy maintenance is strictly dependent on a finely regulated fetal-maternal crosstalk in which balanced physical and metabolic modifications and adaptations occur. Of note, adverse external events during the embryonic and fetal development may negatively affect the postnatal health status by altering gene expression and phenotype, a concept referred as the “Barker hypothesis”^10,11^. Thus, mother/fetus mutual interactions, as in the genetic/epigenetic mother/child dyad studies (GEMCDS) are strongly advised^12,13^.

Among the several factors involved on fetal-maternal crosstalk, DNA methylation is one of the most relevant. It occurs through the entire reproductive process from gametogenesis, embryonic development, and maternal–fetal regulation that can impact fetal and adult life health^14–18^. Methylation is a universal biochemical process in which methyl groups are covalently linked to different molecular targets, including but not limited to DNA. Methylation-driven gene regulation is heritable and leads to key DNA structural and functional modifications such as histones changes, chromatin remodelling and RNA interference^19^. At the molecular level, methylation is ensured by DNA methyl-transferases encoded by *DNMTs* genes by transferring a methyl group to DNA using the folate cycle as methyl source and balanced by TETs enzymes to reestablish the unmethylated cytosine by active or passive demethylation^19–21^. Folate cycle is finalized via the conversion of S-adenosylhomocysteine (SAH) to S-adenosylmethionine (SAM), considered the universal methyl donor, to the cytosine residue within CpG enriched regions^20^. The availability of one-carbon units is essential for the correct establishment and maintenance of methylome and imprinting, then inadequate availability of methionine or folate may affect epigenetic processes^22^ and gene expression^23^. In addition, DNA methylation levels is influenced by inflammation, cytokines (CKs) and CRP levels^24^ suggesting altered CpG methylation as a consequence of high CRP levels^25^. Direct and indirect associations with global DNA methylation have been demonstrated by abnormal inflammatory reactions and selected SNPs show complex mutual interactions towards inflammation and pregnancy loss.

Long Interspersed Nuclear Elements-1 (LINE-1) are a family of related class I transposable elements, one of the most successfully integrated mobile element in the human genome accounting for about 18% of the human genome. Although LINE-1 is widely present within the mammal genome, among the hundreds of thousands copies, only thousands contain 5'UTR, so LINE-1 methylation may represent in part the whole genome status. Methylation of another repeat-element (Alu) may represents global methylation more than LINE-1 because there are millions of copies among mammal genome. In practice, LINE-1 methylation status is commonly considered a valuable surrogate of global DNA methylation^26^ and it has been recently assessed in combination with telomere length as a predictor of successful IVF or as a potential mechanism for pregnancy maintenance^27–29^. Accordingly, events of epigenetic regulation in LINE-1 retrotransposition play crucial roles during embryogenesis, early fetus development, and adult life. LINE-1 hypomethylation has been associated with pathogenesis of several complex diseases as in the case of neural tube defect (NTD) in which lower levels of LINE-1 methylation was found in the placenta of NTD mothers versus controls with NTD risk increased as the level of LINE-1 methylation decreased, furtherly LINE-1 hypomethylation was also associated with a significant increase in expression level of a LINE-1 encoded transcript. These findings have been ascribed to genomic DNA instability and changes in chromatin accessibility caused by hypomethylation^30,31^. LINE-1s can be both intragenic or intergenic, and the insertion of active full-length LINE-1 sequences into the introns of host genes significantly disrupts gene expression, and this has been demonstrated by assessing the expression of genes containing LINE-1 having a higher chance to be repressed both in cancer and hypomethylated normal cells^32,33^. This contributes to senescence and aging processes also in age related complex diseases as recently demonstrated in age related hearing loss or sudden hearing loss and in age related macular degeneration^34–36^. Moreover, maintenance of genome integrity is crucial for embryo development, and epigenetic remodeling during primordial germ cell and fetus evolution may contribute to genome instability since DNA methylation mechanisms are crucial to silence retrotransposons^37^. DNA methylation plays an important role in the suppression of retrotransposon activation during early preimplantation embryo development, since differentiated cells have minimal to null retrotransposition and the expression of retrotransposons or reactivation of LINE-1 might cause pregnancy failure by causing mutations in the host genes by retrotransposition events disrupting in turn the coding regions^28^. Finally, inflammation, oxidative stress, cancer and associated chemo-therapies can epigenetically affect the DNA of gametes, modifying the biological cell age compared to the physiological effects of the chronological aging, and these dangerous epigenomic signatures may have possible transgenerational transmission^38^.

Moreover, genetics and epigenetics interplay is of interest for both those genes directly involved in the methylation processes, and those related to inflammation, immunity, angiogenesis and blood group, widely explored by association studies and meta-analyses suggestive of the existence for inheritance traits.

To investigate whether phenotypic variability in miscarriage was under any hereditary influence, a large study explored genetic and environmental influences on miscarriage rates by a twin study^39^. The authors, by analyzing 3234 female twins equally distributed between monozygotic and dizygotic did not find genetic variation (heritability) as a common cause of miscarriage apart from abnormal embryo karyotype, concluding that women propensity to miscarriage has low heritable basis, though genetic effects might be maintained by a constant and common insertion of novel mutations. On the other hand, single or compound analyses of gene variants support a genetic predisposition in the mother and or in the embryo, and also considering the case of the vanished twin or of the reduced fetal viability for particular coinheritance in *cis* or *trans* of *MTHFR* gene variants, this concept has not been confirmed by other studies that concluded the dizygotic twinning is not associated with *MTHFR* haplotypes^40–42^. On the other hand, a recent report on the genetic architecture of sporadic and multiple miscarriage concludes stating that its complex etiopathogenesis is driven in part by genetic variations mainly related to placental biology, adding that SNP-heritability together with other acquired circumstances may have a larger contribution^43^. Finally, several recent investigations on selected groups of SNPs recognized promising pathways to be further explored.

*MTHFR* gene (1p36.22) encodes a pivotal enzyme in cycling folate isoforms, producing the most active methyl-THF (CH3-THF). *MTHFR* gene variants (i.e., rs1801133, C677T and rs1801131, A1298C) significantly affect the enzyme activity leading to intracellular folate unbalancing affecting the maternal–fetal crosstalk during pregnancy as hypothesized in the GEMCDS^12,13,44^. Association of homocysteine, CRP, fibrinogen and gestational diabetes with EPL and poor pregnancy outcomes have been recently published^45^.

*CRP* gene (1q23.2) encodes a protein involved in the complement cascade activation and amplification. CRP greatly increases during the acute phase or other inflammatory stimuli and is associated with host defense based on its ability to recognize foreign antigens and damaged cells by interacting with humoral and cellular effector systems. High levels are associated to preterm delivery and other complications^46^ and CRP genotype and maternal plasma levels in the first trimester have been also investigated^47^ including rs2808635/rs876538 variants being associated to basal and stimulated CRP levels^48^.

*FGA* and *FGB* genes belong to the fibrinogen cluster (4q32.1-4q31.3), *FGG* gene included. Stimulated by proinflammatory triggers also cooperate in the quality of the 3D-organization of the fibrin scaffold necessary in every heling process and also crucial in the blastocyst implantation and embryo transfer after IVF^49,50^. Associations of gene variants in *FGA* (rs6050) and *FGB* (rs1800790) with pregnancy outcome have been investigated connecting both fibrinogen levels and fibrin architectures^51^.

*F13A1* (6p25.1) and *F13B* (1q31.3) genes have been investigated in complex diseases and in pregnancy loss with controversial results in association or not with fibrinogen levels often considering their main functional gene variants coinherited with the fibrinogen gene cluster polymorphisms^51–53^. Combined investigations merit particular importance because rs5985 and rs6003, respectively in *F13A1* and *F13B* genes, interact with the activation of the FXIIIA2B2 complex and with the fibrinogen gene cluster in both acute inflammation and coagulation phases tuning in turn fibrinogen levels, 3D-fibrin architecture and MMPs resistance in any healing or remodeling phases^35,54–57^.

*TP53* gene (17p13.1) has been widely investigated as tumor suppressor favoring genome stability. Its role in reproductive medicine and placental vasculature has been recently investigated, since many of the steps involved in implantation-apoptosis rate are regulated by p53, moreover the key gene variant P72R (rs1042522) has been studied in pregnancy maintenance, including implantation failure, IVF, and prenatal sex selection^58–62^.

*CFH* gene (1q31.3) has essential role in the complement cascade activation and regulation also crucial for placentation and fetus development^63^. Among the several SNPs of complement cascade genes investigated in pregnancy complications^64^, rs1061170 has been found associated with RPL by uric acid and triglyceride anomalous levels during pregnancy^65^.

*APOE* gene (19q13.32) and *Ɛ4* allele/haplotype (rs7412/rs429358) have been mostly investigated in neurodegeneration and cognitive impairment and recently in the quality of immune response after SARS-CoV2 vaccine^66–68^. Its role in RPL and implantation failure has been investigated as single gene or in combination with selected candidate genes by meta-analysis ascribing to the *Ɛ4* allele the highest risk^69,70^.

*ABO* gene (9q34.2) by the association with ABO blood group has been considered an independent risk factor in the occurrence of pregnancy related complications with different results^71,72^. Interestingly, ovarian capacity and menstrual disorders have been associated to blood groups and the common *ABO* gene variant (rs657152) may be an interesting candidate in the maternal tolerance-rejection processes^73,74^.

Finally, pro- and anti-inflammatory CKs have been largely explored in pregnancy due to their role in the finely regulated immune programming^75^ aimed at conferring the required tolerance to the developing embryo and the appropriate protection against pathogens^76,77^. As a consequence, placental immunology dysfunctions and/or disturbances in specific subsets of maternal immune cells (e.g., natural killer, NK), T-helper unbalance (Th1/Th2) or between regulatory T cells (Treg)^78^ and Th17 are involved in EPL^79–81^. A realistic vision of CKs role is that they do not play independent pathogenic actions, rather they are part of a more complex regulatory network methylation score included^25,82^.

The main aim of the present research is to improve a mere case–control comparison performed by single gene analysis towards a more robust and multilayer tool able to disclose complex mutual interactions hardly recognizable by standard statistical approaches. For these reasons, we investigated the association between global DNA methylation and SNPs of selected genes involved in immune regulation, implantation-apoptosis, angiogenesis, genomic stability, together with selected inflammatory markers (i.e. CKs) as key factors affecting the risk of EPL and RPL. To highlight potential interactions, we included the variable-reduction methods based on the principal components analysis (PCA).

## Material and methods

### Study design and samples collection

A retrospective study aimed at assessing inherited/acquired predispositions to EPL has been performed in a cohort of 230 pregnant women by comparing those who experienced spontaneous miscarriage (EPL, n = 123) with a group of pregnant who underwent voluntary interruption according to the Italian law, 194, Art. 6 comma b (VPI controls, n = 107) referring to the Hospital-University of Ferrara, Italy. The study involving human participants was reviewed and approved by the local regional ethical committee, the participants provided their written informed consent to participate in the study. The following exclusion criteria have been considered: (1) concomitant infections; (2) immune deficiency condition or immunosuppressive treatments; (3) inherited predispositions to abortion; (4) severe uterine malformation; (5) endocrine unbalancing and (6) accidental intake of teratogenic drugs. Finally, close relative patients have been excluded from the study.

Population characteristics for the whole group and after stratification by EPL-cases and VPI-controls are shown in Table 1. Participants had a gestational age ≤ 12 weeks and underwent to whole blood draw, plasma samples were processed within 2 h from drawing blood, and they were frozen at − 80 °C in multiple aliquots and blind tested. DNA was isolated from frozen whole blood by using an automated DNA extraction and purification robot (BioRobot EZ1 system, Qiagen; Hilden, Germany).

**Table 1:** Demographic and clinical data of EPL and VPI control groups.

	Whole cohort (n = 230)	EPL cases (n = 123)	VPI controls (n = 107)
Age, median (IQR)	34 (28–39)	36 (31.5–39)	32 (26.5–35)
Parity, n (%)			
0	121 (52.6)	88 (71.5)	33 (30.8)
≥ 1	109 (47.4)	35 (28.5)	74 (69.2)
Weeks of gestation (mean ± SD)	9.85 ± 1.52	10.01 ± 1.12	9.7 ± 1.78

### Genotyping

Detection of the selected gene variants was as follows: *F13A1 (V34L; rs5985; G* > *T), F13B (H95R; rs6003; C* > *T), FGA (T312A; rs6050; T* > *C), FGB (rs1800790;* −455*G* > *A), TP53* (P72R; rs1042522; C > G), by rhAmp SNP genotyping technology (IDT, Integrated DNA Technologies, Coralville, IA, United States) on the QuantStudio3 Real-Time PCR System (Thermo Fisher Scientific, USA), as previously described^67,83^; *CFH* (Y402H; rs1061170; C > T), *CRP* (rs2808635; G > T; rs876538; T > C), *ABO* (rs657152; A > C), *MTHFR* (A223V; rs1801133, C > T; E429A; rs1801131; A > C), *APOE* (R158C; rs7412, C > T; C112R; rs429358, T > C), by pyrosequencing (Pyromark ID System, Biotage, AB, Uppsala, Sweden) after standard PCR on Agilent SureCycler 8800 (Agilent Technologies, Santa Clara, CA, USA) as previously described^67^. DNA samples with known genotype were used as internal control references for all the sequencing, and a random number of samples (15% for each genotype) were reanalysed as internal quality control as previously described^84,85^.

### LINE-1 methylation by pyrosequencing

Extracted DNA (500 ng) from each sample (DNA isolation Qiagen, Hilden, Germany), was bisulfite-converted by EpiTect 96 Bisulfite Kit (Qiagen, Hilden, Germany), according to the manufacturer’s recommendation and 50uL of converted DNA was stored at − 20 °C. The long interspersed nucleotide element 1 (LINE-1) was analysed as surrogate of genome DNA methylation. CpGs sites (+ 306 to + 364; GenBank accession number: X58075) were PCR amplified and then analysed by PyroMark Q96 ID (Qiagen). A 150 bp amplicon of the LINE-1 sequence was amplified by Pyromark PCR kit (Qiagen) and specific LINE-1 primers (Fw: 5’-TTTTGAGTTAGGTGTGGGATATA-3’; Rev: 5’Bio-AAAATCAAAAAATTCCCTTTC-3’; Seq: 5’-AGTTAGGTGTGGGATATAGT-3’), on the SureCycler_8800 (Agilent Technologies, Mulgrave, AU). Thermo-cycling protocol was as follows: one initial step 95 °C, 15 min; followed by 38 cycles of 94 °C, 30 s; 55 °C, 30 s; 72 °C, 30 s; plus, final 10 min extension at 72 °C. PCR specificity was verified by 8.5% PAGE. Methylation of CpG dinucleotides was calculated as the percentage of cytosine nucleotides relative to the sum of cytosine and thymine nucleotides in a given position by Pyromark Q96 software v1.01. Overall LINE-1 DNA methylation was calculated as the mean of the C percentage of the CpGs sites analysed.

### Inflammatory CKs analysis in plasma samples

Frozen plasma samples were analysed by the MILLIPLEX MAP Human Cytokine/Chemokine high sensitivity panel (Merck Millipore, Billerica, MA) to simultaneous quantify the following human CKs: IL6, IL10, IL17A, IL23. Samples were processed according to manufacturer's protocols and read on a MAGPIX instrument^86^ equipped with the MILLIPLEX-Analyst Software using five-parameter nonlinear regression formula to compute CKs concentrations from the standard curves as previously described^87,88^.

### Statistical analysis

Statistical analyses were performed using SPSS Statistics Version 22 (SPSS Inc., Chicago, IL, USA) and MedCalc version 20.112 (MedCalc Software Ltd., Ostend, Belgium). All figures were produced by GraphPad Prism9 (GraphPad Software, Inc., San Diego, California USA), unless otherwise specified. The Kolmogorov–Smirnov test was used to verify variables normal distribution. Normally distributed data are presented as mean and SD, while non-normally distributed data are presented as the median and interquartile ranges (IQR). Student’s t-test was to compare differences in normal variables between two independent groups and Mann–Whitney U test for non-normal variables. Pearson’s test was used to assess correlation analyses. Crude ORs calculation and 95% CI have been applied in single SNP analyses and Bonferroni correction for multiple SNPs comparisons has been utilized.

Genotypes, methylation, CKs concentration and age were subjected to PCA. SNPs were scored 1, 2, 3 to represent common homozygous, heterozygous, and rare homozygous variant respectively, to indicate an increasing copy number of the variant allele (i.e., 0, 1, 2 respectively). As regards *CRP* and *MTHFR* variants, when not specified the gene symbols account for both variants analysed, unless otherwise specified (i.e., *CRP(1)* accounts for rs876538; *CRP*(2) accounts for rs2808635; *MTHFR(1)* accounts for rs1801133; *MTHFR(2)* accounts for rs1801131); finally, *APOE* (rs7412/rs429358) accounts for ε3/ε4 haplotypes as previously described^67^. Age, methylation and CKs were centred and scaled before PCA according to the formula (*x*-value—mean value)/SD; [Z = (x – μ)/σ]. Collinearity diagnostic evaluation was assessed by variance inflation factor and values below 5.0 have been considered as threshold. PCA was performed by retaining those PCs with Eigenvalue exceeding 1.0. Eigenvector of independent variables with absolute value exceeding 0.3 (+ or −) was included. Variables with a loading above the cut-off point 0.3 were considered to be dominant in a component. Scores for each PC for each individual were extracted by using regression models. Retained PCs were computed in logistic regression analysis for presence/absence of EPL (Yes = 1, No = 0) *versus* PCs. *P*-values were two-sided with threshold for statistical significance fixed to *P* ≤ 0.05.

### Ethics approval and consent to participate

This study was approved by the Ethics Committee of the Hospital-University of Ferrara, Italy (Protocol n. 91-2013, 13/11/2014; PRUA1GR-2013-00000220), samples were collected after the patient signed an informed consent form according to the Declaration of Helsinki, all relevant ethical regulations were followed.

## Results

### LINE-1 DNA methylation

Population group and subgroups are as shown in Table 1. LINE-1 mean methylation in EPL was significantly lower than in VPI controls (81.34 ± 4.66 *vs* 85.82 ± 3.65; respectively; *P* < 0.00001). Intra case analysis ascribed to the RPL subgroup the lowest mean methylation level when compared to the remaining EPL subgroup (80.39 ± 4.29 *vs* 82.48 ± 4.87 respectively; *P* = 0.001). In addition, the different mean age in the cases and control group did not account for changes in the statistical significance of the comparison when the test was corrected for age (adjusted *P*-value = 0.0001).

**Figure 1:**
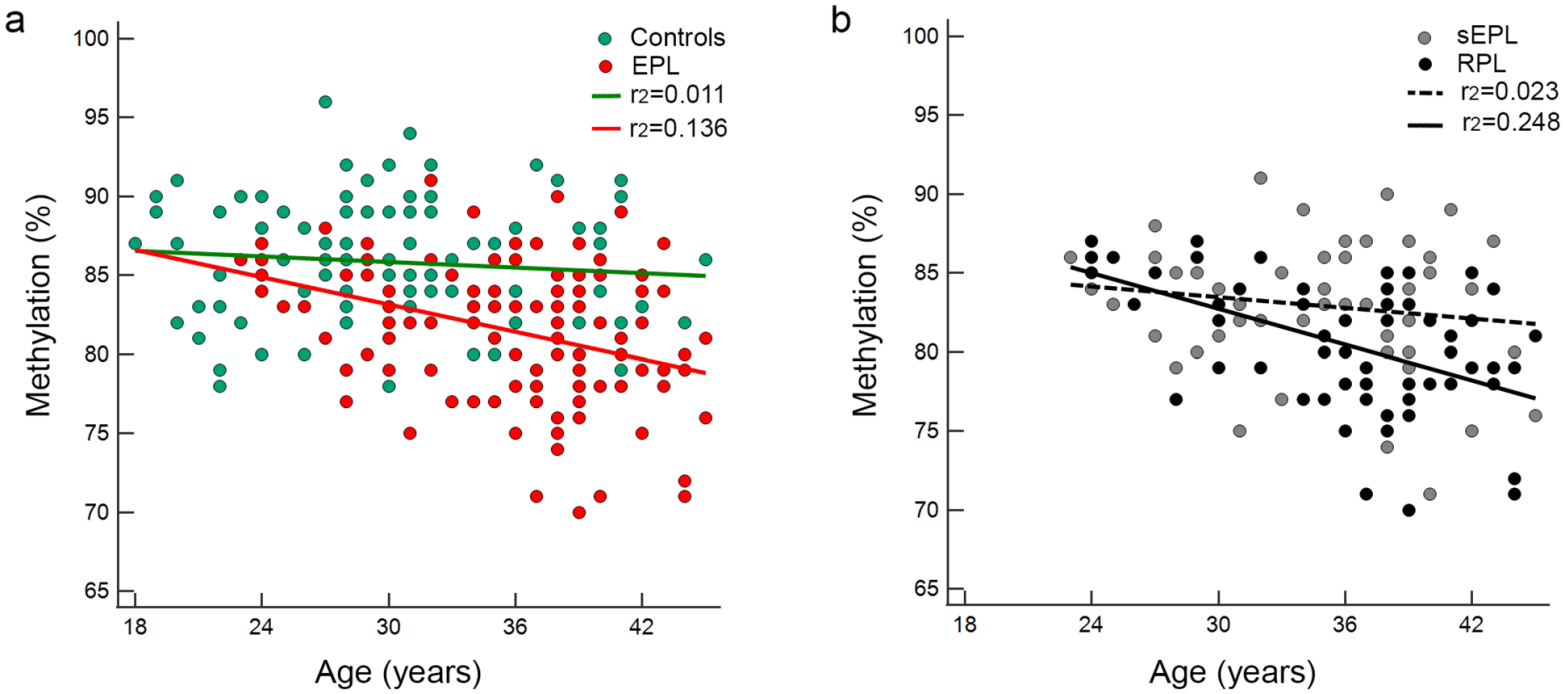
Methylation-age correlation analysis. (**a**) Correlation between methylation and age distribution in the whole cohort stratified by VPI controls (green dots) and EPL cases (red dots). Regression lines are shown (green line and red line for VPI controls and EPL respectively). (**b**) Correlation between methylation and age distribution in the EPL group stratified by single event (non-recurrent) cases (sEPL, grey dots) and recurrent EPL (RPL, dark dots). Regression lines are shown (continuous and dotted line for RPL and sEPL respectively). Each panel shows the *r*^2^-coefficient for the regression lines.

As shown in Fig. 1a, a negative correlation existed between methylation levels and age, and it was stronger among the whole EPL cases than VPI controls (*r*^*2*^ = 0.136 *vs. r*^*2*^ = 0.011 respectively) as well among RPL subgroup when compared to the remaining EPL subgroup (*r*^*2*^ = 0.248 *vs. r*^*2*^ = 0.023 respectively) (Fig. 1b). Moreover, by stratifying the age-matched subgroups (EPL cases *vs* VPI controls) both the mean methylation level comparison and the age-methylation correlations kept trends in favor of the control group, confirming that regardless different mean age, EPL cases had significantly lower mean methylation levels (*P* = 0.0001) and higher age-dependent methylation declining (*r*^*2*^ = 0.140 *vs. r*^*2*^ = 0.033 respectively).

Interestingly, the same subanalysis (i.e. age *vs* methylation) stratified by *MTHFR* genotype, ascribed to the T-677 dysfunctional polymorphic allele a more robust negative correlation with age either by comparing the opposite genotypes in the whole cohort (*r*^*2*^ = 0.123 *vs r*^*2*^ = 0.212 respectively in 677C-carriers and 677TT-genotype; P = 0.025), or by comparing the TT-genotype in EPL *versus* VPI subgroup (*r*^*2*^ = 0.283 *vs r*^*2*^ = 0.057 respectively; P < 0.01) (Supplementary Fig. 1).

### Single gene analyses

Table 2 shows the EPL risk calculation (crude OR and *P*-values) according to the genetic model applied and the considered subgroup of cases (see Supplementary Table 1 for the complete dataset). All the SNPs investigated were in Hardy–Weinberg equilibrium except for *CFH*_rs1061170_ (*X*^2^ = 6.852) and *ABO*_rs657152_ (*X*^2^ = 7.064).

**Table 2:** EPL risk calculation (OR) in selected genes.

Gene	Genetic model OR(CI;95%); *P*			
	(+ + / + −) *vs.* − −	+ + *vs.* (+ − / − −)	+ + *vs.* − −	+ *vs.* −
***F13A1***	–	0.24(0.065–0.9)	0.23(0.06–0.88)	0.60(0.37–0.98)
rs5985		**0.03**	**0.03**	**0.04**
***F13B***	–	–	–	0.5(0.28–0.88)
rs6003				**0.015**
FGA*	–	–	–	0.58(0.33–1.0)
rs6050				**0.05**
FGB**	1.97(1.01–3.87)	–	–	–
rs1800790	**0.045**			
CRP**	–	0.15(0.014–0.87)	–	–
rs2808635		**0.035**		
ABO*	–	0.23(0.06–0.82)	–	–
rs657152		**0.02**		
***MTHFR***	2.03(1.2–3.47)	–	–	1.6(1.09–2.33)
rs1801133	**0.009**			**0.015**
***TP53***	0.54(0.32–0.92)	–	–	0.65(0.43–1.00)
rs1042522	**0.03**			**0.05**

Homozygotes for the common and polymorphic (rare) allele are indicated as −− and ++ respectively; heterozygotes are indicated as +−. Genetic models applied: dominant [(++/+−) *vs.* −−]; recessive [++ *vs* +−/−−]; opposite homozygotes [++ vs −−]; allele comparison [+ vs. −]. Genetic models were used for ORs computation as described in the “Methods” section.

Significant *P*≤0.05 are shown in bold.

* and ** indicate RPL and non-recurrent EPL respectively. Bonferroni correction for multiple comparisons did not reach significant outputs for the SNPs investigated.

*F13A1* rs5985 genotypes were differently distributed between whole cases and controls (*P* = 0.069), reaching significant results in the alleles frequency comparison (*P* = 0.04). The under-representation of the T-allele in the case group yielded OR-values below the unit value for both genotype recessive model (OR = 0.24; 0.065–0.90; *P* = 0.03) and allele (OR = 0.60; 0.37–0.98; *P* = 0.04) comparisons. This accounted for a protective effect against EPL of more than 4-folds in TT-homozygous women.

*F13B* rs6003 genotypes were differently distributed between whole cases and controls (*P* = 0.07), reaching significant results in the alleles frequency comparison (*P* = 0.015). The under-representation of the C-allele in the case group yielded OR-values below the unit value for both genotype recessive model (OR = 0.23; 0.04–1.1; *P* = 0.07) and allele (OR = 0.50; 0.28–0.88; *P* = 0.015) comparisons. This latter accounted for a protective effect against EPL of about 2-folds in C-carrier women. The protective effect was even higher (i.e. 3-folds) in the RPL subgroup (OR = 0.33; 0.13–0.87; *P* = 0.025).

*FGA* rs6050 genotypes were similarly distributed in the whole cases and controls, reaching borderline significant results by comparing the allele frequency in the RPL subgroup (OR = 0.58; 0.33–1.0; *P* = 0.05).

*FGB* rs1800790 genotypes were similarly distributed in the whole cases and controls, though appreciable results were found in both allele (OR = 1.7; 0.98–2.98; *P* = 0.06) and genotype dominant model (OR = 1.97; 1.01–3.87; *P* = 0.045) comparisons in the younger subgroup of cases. This latter accounted for an increased EPL risk of about 2-folds in A-carrier women.

*CRP* rs2808635 genotypes were similarly distributed between whole cases and controls, though significant results were found in the younger subgroup of cases both in genotype distribution (*P* = 0.03) and recessive model (OR = 0.15; 0.014–0.87; *P* = 0.035) comparisons. This latter accounted for an increased EPL risk of more than 6-folds in G-carrier women.

*ABO* rs657152 genotypes were similarly distributed between whole cases and controls (*P* = 0.08). Nonetheless, the under-representation of the TT-genotype in the case group yielded OR-values below the unit value in the genotype recessive model comparison (OR = 0.48; 0.22–1.08; *P* = 0.07). The protective effect was even higher in the RPL subgroup (OR = 0.23; 0.06–0.82; *P* = 0.02). This latter accounted for a protective effect against EPL of more than 4-folds in TT-homozygous women.

*MTHFR* rs1801133 genotypes were differently distributed between whole cases and controls (*P* = 0.03). The over-representation of the T-allele in the case group yielded increased risk values in both genotype dominant model (OR = 2.03; 1.2–3.47; *P* = 0.009) and allele (OR = 1.6; 1.09–2.33; *P* = 0.015) comparisons. The risk effect was even higher in the younger subgroup of cases (OR = 2.94; 1.44–6.01; P = 0.003).

*TP53* rs1042522 genotypes were similarly distributed between whole cases and control groups (*P* = 0.069). Nonetheless, the under-representation of the G-allele in the case group yielded OR-values below the unit value in the genotypes dominant model comparison (OR = 0.54; 0.32–0.92; *P* = 0.03). The protective effect was even higher in the younger subgroup of cases (OR = 0.44; 0.23–0.88; *P* = 0.02). This latter accounted for a protective effect against EPL of more than 2-folds in the G-allele carrier women.

EPL risk calculation (crude OR and *P*-values) for the above mentioned SNPs is summarized in Table 2, after Bonferroni correction they did not reach significant outputs.

The remaining SNPs in *CFH* (rs1061170) and in *APOE* (rs7412; rs429358) genes did not yield significant results by single analysis, but they were included in the PCA multilayer exploration.

### Serum cytokine profile

**Figure 2:**
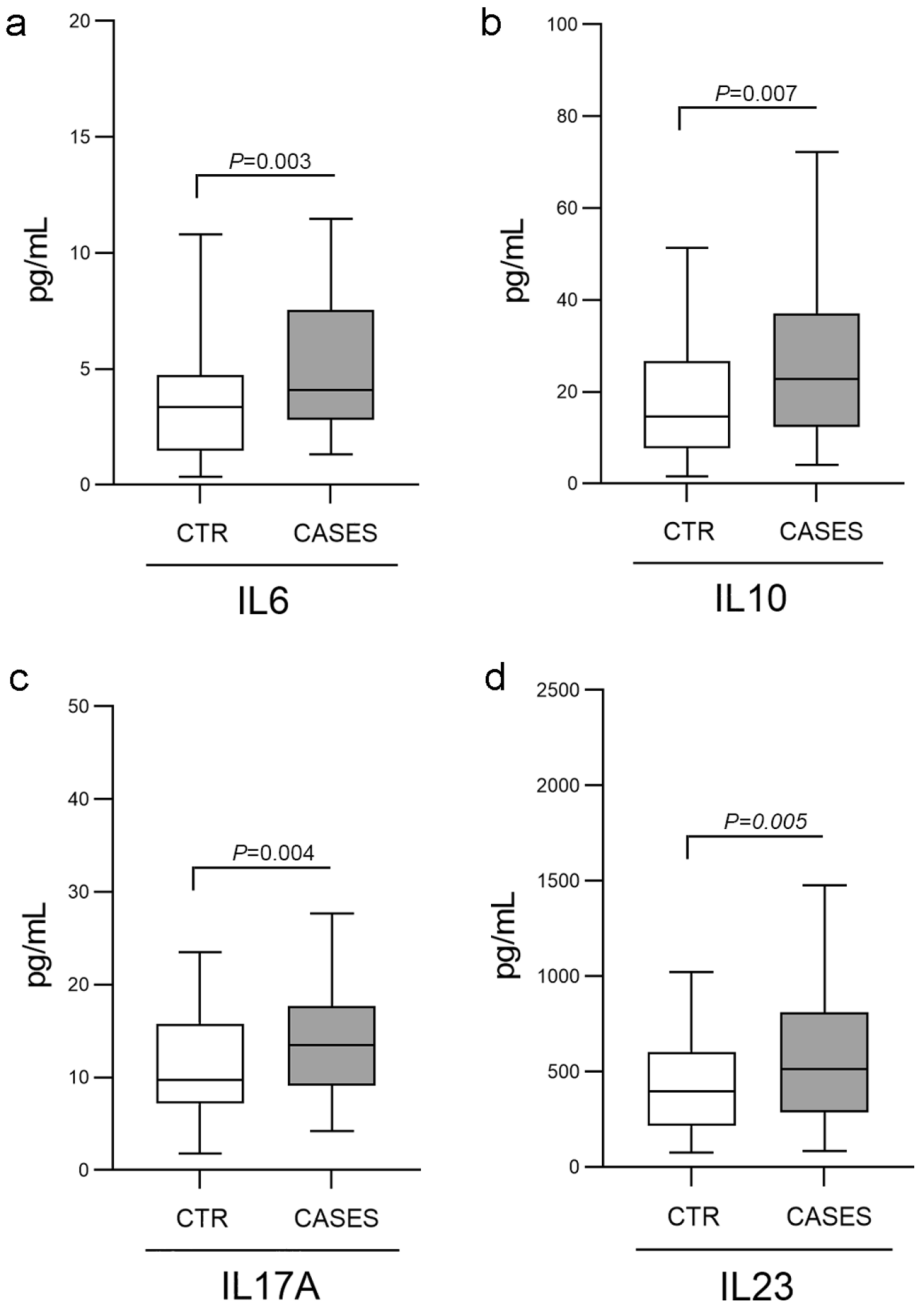
CKs levels distribution. Circulating IL6 (**a**), IL10 (**b**), IL17A (**c**) and IL23 (**d**) levels in VPI controls and EPL cases. Box plots show median and IQR. *P* values are indicated on top of each panel.

Figure 2 shows the mean circulating levels of IL6, IL17A, IL23 and IL10 significantly higher in the whole case group than in the controls (P < 0.0001). By comparing RPL versus the remaining EPL cases no statistical differences have been observed (data not shown).

**Figure 3:**
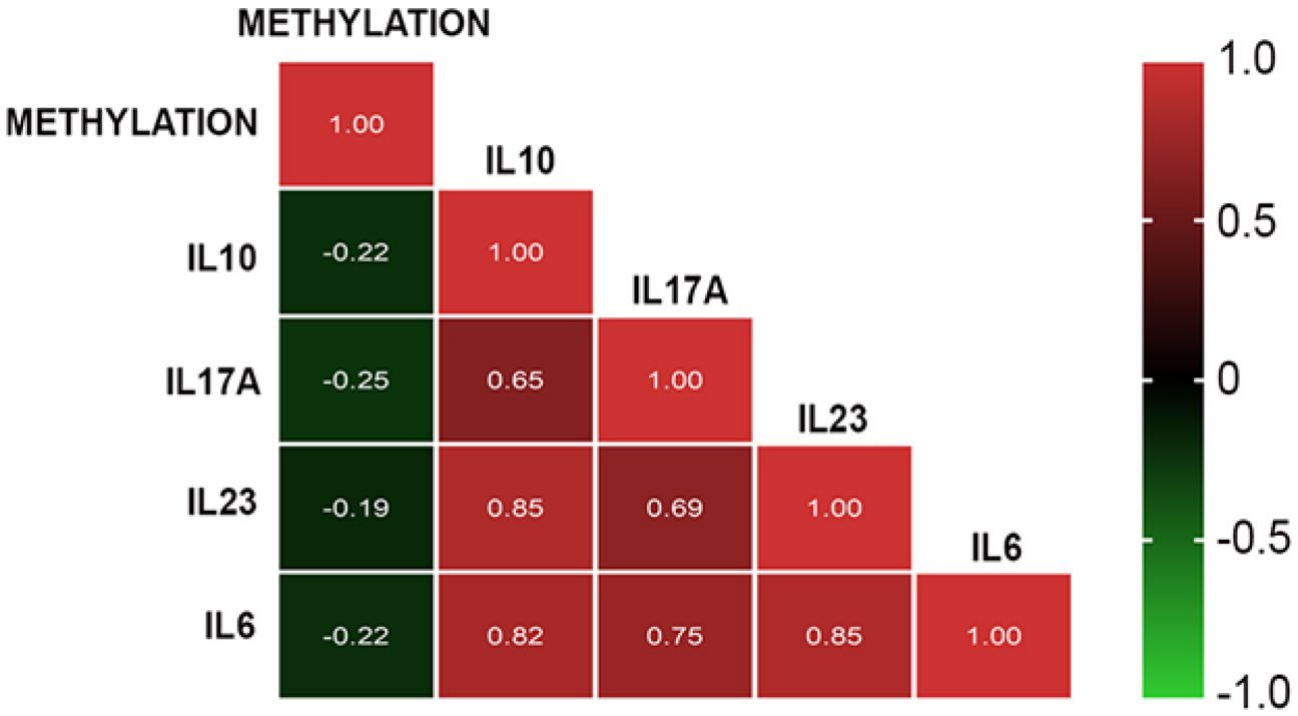
Pearson correlation heatmap between methylation and CKs in the whole group. Red and green indicate a positive and a negative association, respectively. Colour intensity represents the strength of the correlation.

Interestingly, strong inverse correlations have been obtained between each single CK mean level and mean methylation level in the whole group of 230 pregnant women (Fig. 3). Correlation was lost in the RPL subgroup and retained in the remaining EPL cases.

### PCA and logistic regression analysis of the principal components (PCs)

**Figure 4:**
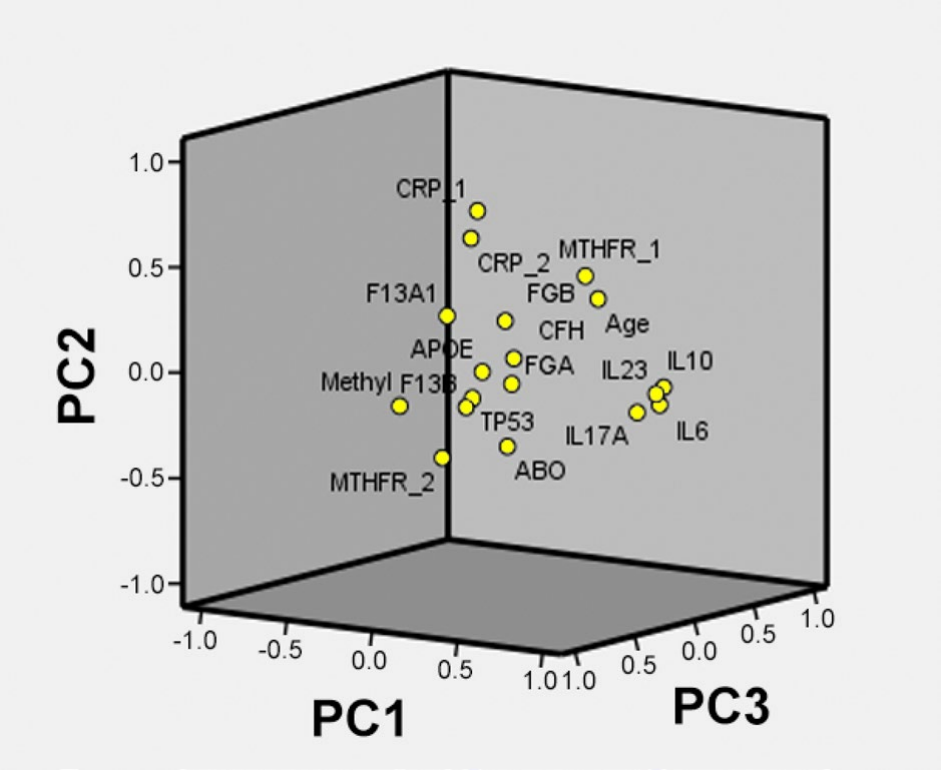
Principal component analysis result for the computed 18 variables: PC1, PC2 and PC3 loadings. Abbreviations: CRP_1 (rs876538); CRP_2 (rs2808635); MTHFR_1 (rs1801133); MTHFR_2 (rs1801131); APOE (rs7412/rs429358) accounts for ε3/ε4 haplotypes; Methyl: methylation. Plotted by SPSS (Statistics Version 22).

**Figure 5:**
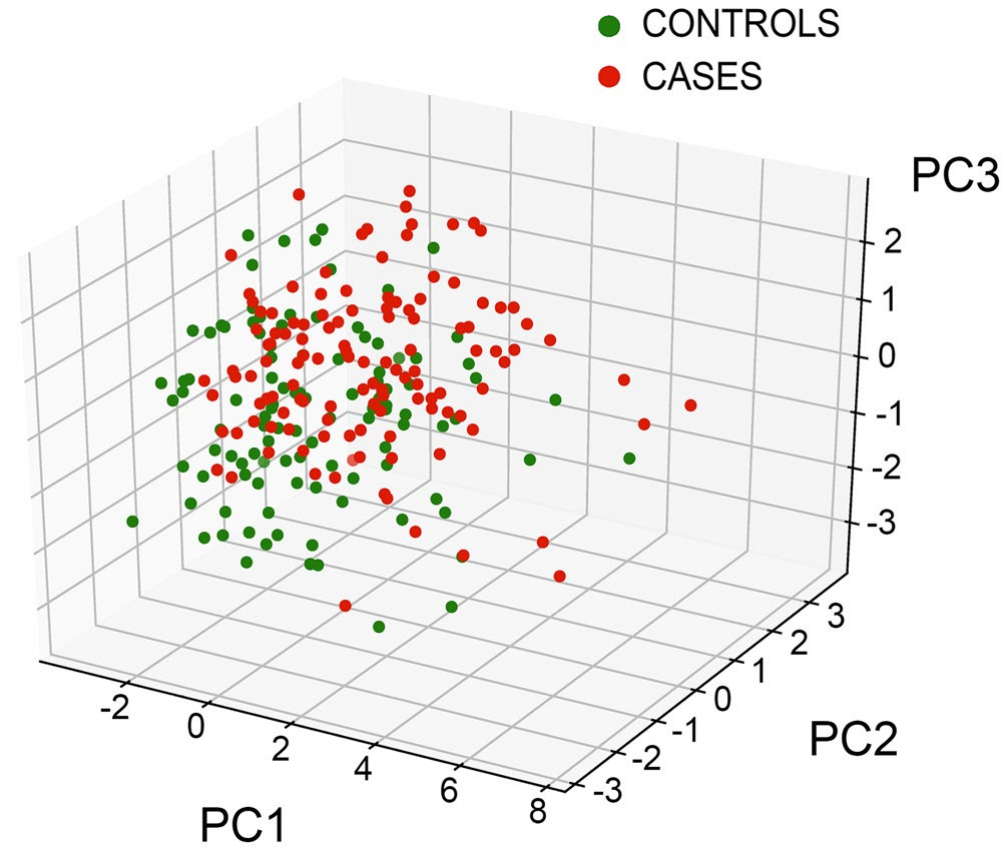
3D-loading plot of the scores of the whole cohort (n = 230) based on PC1, PC2 and PC3. Red dots: n = 123 EPL; green dots: n = 107 VPI controls. Plotted by bioinformatics.com.cn/srplot.

We performed PCA and logistic regression analysis to explore relationships between the significant PCs and the risk of EPL. PCA was completed with all the 18 variables and the first 7 PCs have been retained (i.e., eigenvalue > 1.0) explaining approximately 65.8% of the total variation. 3D-loading plots show how all the 18 computed variables allocate (Fig. 4) and how the whole group of 230 cases stratifies (Fig. 5) along with the first three selected PCs overall explaining about 40% of dataset intergroup variance.

Considering those eigenvectors of independent variables with absolute value exceeding 0.3, the selected 7 PCs mainly accounted for: PC1 (*F13A,* methylation, IL6, IL10, IL23, IL17A); PC2 (*CRP, ABO, MTHFR*); PC3 (*CRP*, *MTHFR*, age, methylation); PC4 (*F13B, FGA, FGB, APOE, TP53*, age, methylation); PC5 (*F13B, FGA, MTHFR, TP53*); PC6 (*F13A, CFH, ABO, MTHFR, TP53,* age) and PC7 *(FGB, CFH, ABO, APOE*) as summarized in Table 3. By including EPL (Y/N) as dependent variable and PCs as the independent variables in a logistic regression model, we found significant positive association with EPL risk in PC1 and negative association with EPL risk in PC3, PC4 and PC6. In detail, the contribution of the significant PCs was: PC1 (19.8%); PC3 (9.6%); PC4 (7.6%), PC6 (6.4%), (see Table 4).

**Table 3:** Loadings of Principal Components in the whole cohort (n = 230).

Variables	PC1	PC 2	PC 3	PC 4	PC 5	PC 6	PC 7
*F13A1 (rs5985)*	** − 0.317**	0.193	0.031	− 0.294	− 0.211	**0.506**	0.130
*F13B (rs6003)*	− 0.026	− 0.172	0.287	** − 0.325**	**0.634**	− 0.035	0.144
*FGA (rs6050*	0.068	− 0.090	0.038	**0.601**	**0.329**	− 0.117	− 0.110
*FGB (rs1800790)*	− 0.028	0.188	− 0.043	**0.414**	− 0.124	0.082	**0.473**
*CFH (rs1061170)*	0.204	0.070	0.216	0.005	− 0.184	**0.395**	** − 0.454**
*CRP (rs876538)*	0.126	**0.791**	**0.409**	0.048	0.011	− 0.194	− 0.088
*CRP (rs2808635)*	0.201	**0.691**	**0.569**	− 0.007	0.052	− 0.115	− 0.066
*ABO (rs657152)*	0.157	** − 0.352**	0.202	0.231	0.187	** − 0.317**	** − 0.365**
*MTHFR (rs1801133)*	0.049	**0.328**	** − 0.605**	0.181	0.275	0.146	− 0.020
*MTHFR (rs1801131)*	− 0.087	** − 0.402**	**0.400**	− 0.125	** − 0.524**	** − 0.324**	− 0.019
*APOE (rs7412/rs429358)*	− 0.001	− 0.018	0.186	** − 0.324**	0.149	− 0.271	**0.517**
*TP53 (rs1042522)*	− 0.030	− 0.140	0.229	** − 0.361**	**0.418**	**0.346**	− 0.204
Age	0.190	0.247	** − 0.510**	** − 0.326**	0.043	** − 0.389**	− 0.082
Methylation	** − 0.316**	− 0.175	**0.428**	**0.417**	0.124	0.247	0.281
IL10	**0.911**	− 0.030	− 0.030	0.077	− 0.021	0.054	0.051
IL17A	**0.817**	− 0.145	0.057	− 0.102	− 0.040	0.107	0.054
IL23	**0.917**	− 0.051	0.040	0.023	− 0.051	0.112	0.105
IL6	**0.922**	− 0.103	0.020	− 0.019	0.004	0.080	0.165

In bold the main loadings exceeding the absolute cut-off value > 0.30.

**Table 4:** Principal Component regression analysis in the whole cohort (n = 230).

PCs	OR (95%CI)	*P*	Main loadings of PCs
PC1	1.805 (1.330–2.449)	**0.000**	*F13A1,* Methylation, CKs
PC2	1.283 (0.984–1.674)	0.066	*CRP, ABO, MTHFR*
PC3	0.489 (0.362–0.660)	**0.000**	*CRP, MTHFR,* Age, Methylation
PC4	0.722 (0.551–0.946)	**0.018**	*F13B, FGA, FGB, APOE, TP53,* Age, Methylation
PC5	0.766 (0.585–1.002)	0.052	*F13B, FGA, MTHFR(2), TP53*
PC6	0.612 (0.462–0.811)	**0.001**	*F13A1, CFH, ABO, MTHFR(2), TP53,* Age
PC7	0.887 (0.683–1.151)	0.292	*FGB, CFH, ABO, APOE*

In bold significant *P* values.

Considering that CKs resulted as the strongest components of the major PC (i.e., PC1) and that CKs levels were available for about 85.5% of the whole cohort, we recalculated PCA by excluding those cases lacking CKs assessment. PCA analysis yielded 8 PCs with eigenvalue > 1.0 that explained approximately 72% of the total variation. Considering those eigenvectors of independent variables with absolute value exceeding 0.3, the selected 8 PCs mainly accounted for: PC1 (*F13A,* methylation, IL6, IL10, IL23, IL17A); PC2 (*CRP, MTHFR,* age, methylation); PC3 (*CRP, ABO*, *MTHFR, TP53*); PC4 (*F13B, FGA, FGB, APOE, TP53*, age, methylation); PC5 (*F13A, CFH, MTHFR, TP53,* age, methylation); PC6 (*F13A, F13B, FGA, CFH, MTHFR, APOE*), PC7 (*FGB, CFH, ABO*) and PC8 (*ABO, APOE, TP53*) as summarized in Supplementary Table 2. By including EPL (Y/N) as dependent variable and PCs as the independent variables in a logistic regression model, we found significant positive association with EPL risk in PC1, PC3 and PC4 and negative association with EPL risk in PC2, and PC5 (see Supplementary Table 3). In detail, the contribution of the significant PCs was: PC1 (20.0%); PC2 (10.1%); PC3 (9.3%); PC4 (8.01%); PC5 (6.7%).

## Discussion

Pregnancy loss is a challenging area of the reproductive medicine in which the maternal–fetal crosstalk initiates a series of complex biochemical and cellular interactions in large part genetically and epigenetically compelled^89–91^. According to the fetal origin of adult disease (FOAD), as well as the theory of the developmental origins of health and disease (DOHaD), maternal genetics and epigenetics burden have a great part. In utero and periconceptional exposures to environmental factors may act on genetic predispositions leading to pathological outcomes as pregnancy loss, and among the mechanisms linking environment and genetics, epigenetics (via methylome changes) plays a key role^16,17^. EPL pathogenesis is not fully understood lacking in large part a causative recognition. To increase knowledge in this field, our approach assessed epigenetic, genetic, and biochemical investigations in a well characterized cohort of 230 pregnant women by single analyses and multilayer PCA approaches.

In general, reduction of LINE-1 methylation can be linked to reduced methyl-donor availability via one-carbon metabolism^16^. High methyl groups availability does not necessarily result in increased LINE-1 methylation; a proper channelling of methyl groups in the DNA methylation path is directly dependent on DNMTs enzyme activities and the several one-carbon metabolism enzymes ultimately represented by *MTHFR*^44,92^. Both groups of genes are highly polymorphic and functional gene variants can significantly alter direct associations as recently demonstrated in complex phenotypes as cancer, maternal LINE-1 methylation in Down syndrome, and in type 2 diabetes patients with pre-symptomatic dementia^93–95^. All these mechanisms led to DNA damage and instability reducing faithful DNA synthesis and promoting cell senescence also favouring detrimental LINE-1s activation and in turn aberrant host gene expression. A part, drastic effect as embryo death, abnormal epigenome can influence the onset of infant complex diseases as paediatric cancers or neuro neurodevelopmental diseases in which genetic-epigenetic mother–child dyad has a role (GEMCDS-Study)^12,13^.

The main result of our study is a clear progressive global methylation reduction found in spontaneous miscarriages compared to normal pregnant controls, in which RPL cases also showed the lowest methylation levels when compared either to controls or single pregnancy loss cases. Furthermore, the age-matched subgroup comparison ascribed to cases a significantly stronger age-effect on the lowering grade of the methylation trend, further confirmed in the intra-cases analysis, suggesting a basic dysregulation of the epigenetic mechanisms essential for the maintenance of a healthy pregnancy^27,76^. In this line, correlations between epigenetic clocks and anti-Müllerian hormone or ovarian reserve or successfully IVF have been reported^27,96,97^ suggesting that accelerated epigenetics mechanisms might determine the pregnancy outcome^98^. Abnormal in utero methylation setting may led to early embryo death, since DNA methylation greatly influences early embryo development and trophoblast proliferation assisting spiral artery remodelling essential for embryo implantation and maintenance of an effective maternal-foetal crosstalk^29^. Balanced de novo DNA methylation is not only critical during placentation but also for embryo survival as very recently demonstrated due to a critical role of DNMT3B action^99^. Accordingly, extensive maternal health during pregnancy, including a balanced methylation status and appropriate methyl groups availability, may have permanent impacts on the future health of descendants via global or specific epigenetic mechanisms. Noteworthy, a more robust negative correlation between age and methylation levels was observed in the *MTHFR* 677TT dysfunctional genotype, and at a greater extent in the spontaneous abortion subgroup supporting the concept that a suboptimal haplotype-driven intracellular methyl-groups availability exists^13,44^, and it may affect embryo survival and pregnancy maintenance.

DNA methylation of imprinted genes, and/or genes directly or not related to methyl groups cycling, uterine immune tolerance, inflammation, neo-angiogenesis, apoptosis, cytokine expression, and lipid or folate metabolism, globally contribute to the kaleidoscope of pregnancy maintenance^100^. On the other hand, many genetic risk factors have been largely investigated, mainly by SNPs analysis and meta-analyses utilized for risk prediction, often leading to conflicting or partial results^100^.

Among the most investigated variants there are those of the *MTHFR* gene (rs1801133, rs1801131), that play a key role in the availability of active folate isoforms essential for both faithful DNA neo-synthesis and balanced *de-novo* DNA methylation, crucial processes in embryo survival and foetal growth^12,101^. *MTHFR* T-677 allele (rs1801133) causes low intracellular level of 5CH3-THF, the most active isoform for methyl-group unit transfer by DNMTs. Moreover, a direct correlation has been found between global methylation and systemic inflammation assessed by high CRP levels particularly among carriers of the *MTHFR* T-677 allele that causes global hypomethylation in the low folate range and hyperhomocysteinemia also leading to incomplete vasculature and decreased placental transport^102,103^.

On the other hand, *CRP* rs2808635 and rs876538 gene variants modulate the basal and stimulated circulating levels of CRP^48^, and several studies ascribed to these SNPs prognostic pharmacogenomics information on treatment and drug response included the extent of the humoral response after COVID-19 vaccination^67,104–106^. *CRP* genotypes and CRP levels in pregnant women have been widely investigated^15,19,21^ and rs2808635/rs876538 variants are associated to basal and stimulated circulating levels of CRP and inflammation.

Systemic inflammation reflects not only high circulating CRP and CKs levels but also fibrinogen concentration considered one of the reactive-phase molecules particularly important in pregnancy^107^. Fibrinogen is a target of autoimmune reactions and is involved in the generation of a stable 3D-fibrin meshwork necessary for inflammation control. Stability and perfect fibrin architecture also depend on coagulation FXIIIA^108,109^ and complex fibrinogen/FXIIIA haplotypes (*FGA, FGB, FGG, F13A1*) have been found related to CRP levels during acute-phase reactions^110^. For these reasons, the most investigated SNPs (*FGA* rs6050, *FGB* rs1800790, *FGG* rs1049636, *F13A1* rs5985 *F13B* rs6003) should be globally considered and analysed in complex diseases because of common functional (i.e., 3D-Fibrin structure organization) and genetic associations (i.e., Fibrinogen genes cluster; 4q32.1-4q31.3). Accordingly, compound haplotypes investigations might better account for the global risk assessment.

Similarly, *F13A1* and *F13B* genes carry two main functional loci (rs5985, rs6003 respectively) synergistically involved in the catalytic enzyme activation of the FXIIIA2B2 tetramer, not only in the 3D-Fibrin meshwork organization together with FVII/TF complex^111,112^, but also in the novel angiogenesis processes and tissue healing^109,113^ via* TSP1*-inhibition and *VEGF* expression^114^ crucial mechanisms in embryo implantation and pregnancy maintenance^51–53,85^.

Normal embryo growth also needs appropriate trophoblast proliferation and adequate neo-vessel development; therefore, balanced angiogenesis and apoptosis play important roles for cyto-trophoblast development. *TP53* coding product (i.e., p53 protein) gives protection to germinative cells and embryos by LIF-regulation a crucial cytokine helpful in blastocyst successful implantation^115^. A recent metanalysis on the role of *TP53* rs1042522 found associated risk for RPL in women carrying the P72-allele in every genetic model analyzed^116^ and among the recent explanations for this phenomena, reduced apoptosis, impaired placental structure lacking adequate gas and nutrient exchange, and a prolonged arrest of cells in G1-cycle have been proposed as mechanistic causative reasons^62^.

The role of maternal ABO blood group and pregnancy outcome have been extensively investigated and association with hypertension, preterm birth, diabetes, and cardiovascular complications are well known^117^. Recent attention has been done toward *ABO* rs657152 as responsible for maternal tolerance-rejection processes^73,74^ suggesting involvement of the immune response in carrier cases as recently found in the dynamic of circulating antibody levels detected in healthy subjects after anti-SARS-CoV-2 vaccine^67^.

Basically, a single variable analysis just in part can explain the global complex mechanism responsible for EPL, and after Bonferroni correction for multiple comparisons the investigated SNPs did not reach significant outputs. Globally, the causative discussed rationale altogether reminds to unbalanced inflammation, angiogenesis, apoptosis, immunity, and methylation dysfunctions. In an explorative attempt we decided to investigate the same variables in a cumulative statistics approach accounted by PCA to have a more realistic and comprehensive picture as recently reported for inflammatory biomarker profiles and adverse birth outcome^82^ also supported by the correlation we also found between inflammation (i.e., CKs levels) and methylation^25,118^. Interestingly, PCA analyses in the whole group yielded four principal components with different variables clustering, and these were also significantly associated with the risk of EPL as confirmed by further logistic regression analysis. In detail, PC1 mainly explains cytokines, methylation, and coagulation *F13A1*; PC3 is mainly represented by *CRP*, *MTHFR,* age and methylation. Finally, PC4 was strongly characterized by *F13B, FGA*, *FGB* and *TP53*, together with *APOE,* age and methylation, while PC6 was characterized by *F13A*, *CFH, ABO, MTHFR, TP53* and age*.* With regard to PC2 and PC5, thought with borderline significant associations in regression analysis, they yielded interesting outputs: in PC2, *CRP* represented almost all of the component constituents with *ABO* and *MTHFR*, and PC5 was a comprehensive measure of *F13B, FGA, MTHFR,* and *TP53*. Considering the further PCA analysis performed by excluding those few cases in which cytokines have not been assessed, the outputs largely resembled those of the whole group and *CRP* accounted by PC2 reached now stronger significant association in logistic regression analysis.

## Conclusion

The present explorative analysis suggests how a multilayer approach accounting for genetic, epigenetic, and biochemical factors may allow a rigorous EPL risk assessment. This is in line with the multifactorial nature of spontaneous EPL in which the coexistence of different factors may have effects on the final clinical phenotype showing additive/synergic/antagonistic effects finely detectable by multiple analysis procedures. Several questions have been raised, and to better answer them larger population cohorts must be recruited, also by investigating the foetus (epi)genome. Although the maternal (epi)genetics landscape has a protagonist role, the mother-foetal crosstalk is not less important as estimated by the GEMCDS group in other complex diseases in which the *in-utero* origin of the disease has to be considered^12,13^. Recognizing the etiopathogenesis of EPL embraces great promise and will help to identify prognostic biomarkers and efficient therapeutic targets, as well as designing of novel epidrugs, inducing favorable epigenetic modulation to target and modulate the epigenetic pathways.

### Supplementary Information

Supplementary Information.

## Data Availability

The datasets supporting the conclusions of this article are included within the article and its additional supplementary files.
