# Supplementary material for: Epigenetic role of LINE-1 methylation and key genes in pregnancy maintenance
Source: Sci Rep. 2024 Feb 8;14:3275. doi: 10.1038/s41598-024-53737-2 (PMC10853191; doi:10.1038/s41598-024-53737-2)
Supplement: Supplementary file 1 — Supplementary Information. [file 41598_2024_53737_MOESM1_ESM.docx]

**Epigenetic Role of LINE-1 Methylation and Key Genes in Pregnancy Maintenance**

Veronica Tisato^1,2,3,4^*^§^, Juliana A. Silva^1^*, Fabio Scarpellini^5^, Roberta Capucci^6^,

Roberto Marci^1^, Ines Gallo^1^, Francesca Salvatori^1^, Elisabetta D’Aversa^1^, Paola Secchiero^1^,

Maria L. Serino^3^, Giorgio Zauli^7^, Ajay V. Singh^8^ and Donato Gemmati^1,2,3§^

**Supplementary Material**

**Supplementary Figure 1.** Methylation-age correlation analysis stratified by *MTHFR* C677T

**Supplementary Table 1.** SNPs genotype distribution in EPL cases and VPI controls and crude ORs

**Supplementary Table 2.** Loadings of Principal Components (n=196)

**Supplementary Table 3.** Principal Component regression analysis (n=196)


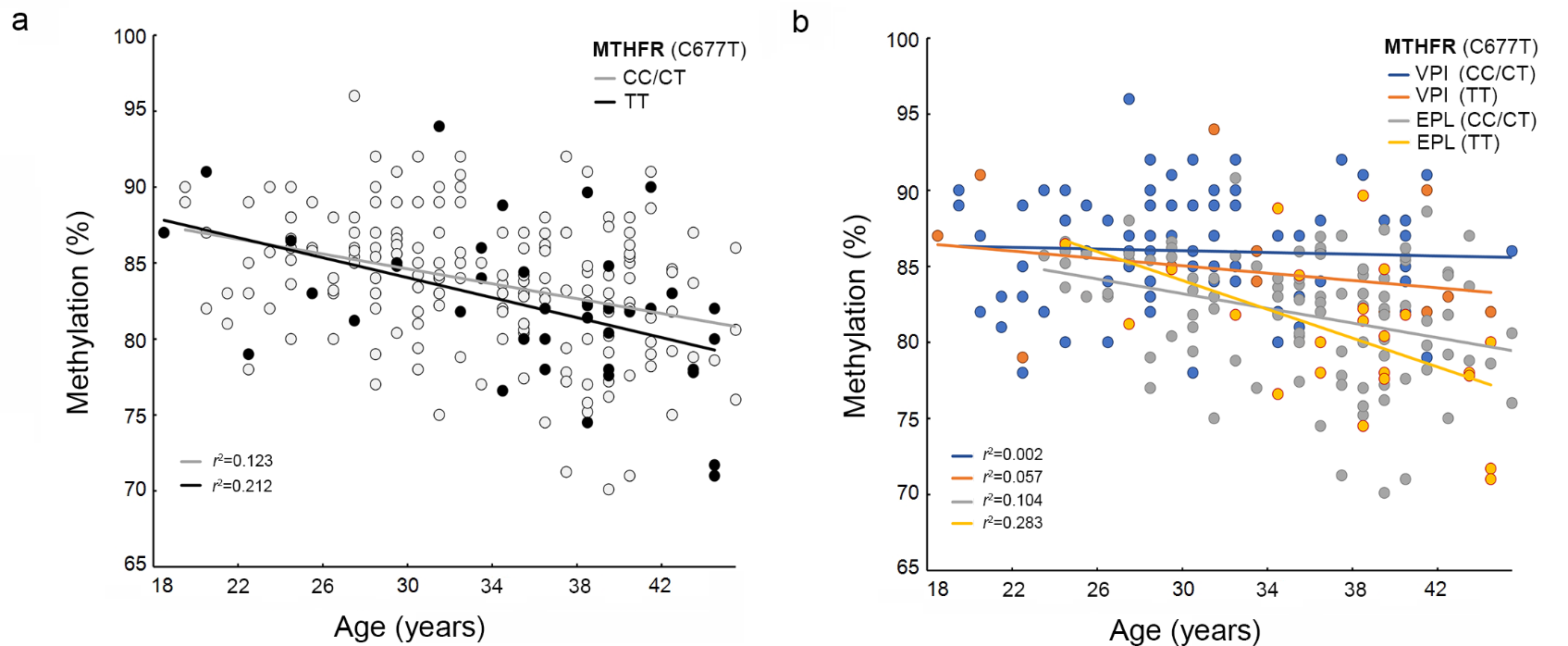


**Supplementary Figure 1. Methylation-age correlation analysis stratified by *MTHFR* C677T.** (**a**) Correlation between methylation and age distribution in the whole cohort stratified by 677C-carriers (grey dots) and 677TT-genotype (black dots). Regression lines are shown (grey line and black line for 677C-carriers and 677TT-genotype respectively). (**b**) Correlation between methylation and age distribution in the EPL *versus* VPI group stratified by 677C-carriers (grey and blue dots for EPL and VPI respectively) and 677TT-genotypes (yellow and orange dots for EPL and VPI respectively). Regression lines are shown (677C-carriers: grey and blue line for EPL and VPI respectively; 677TT-genotype: yellow and orange line for EPL and VPI respectively). Each panel shows the r^2^-coefficient for the regression lines.

| **Supplementary Table 1. SNPs genotype distribution in EPL cases and VPI controls and crude ORs** | | | | | | | | | | | | | | | | | | | |
| --- | --- | --- | --- | --- | --- | --- | --- | --- | --- | --- | --- | --- | --- | --- | --- | --- | --- | --- | --- |
| **EPL cases (n = 123)** | | | | | | | | | | | | | | | | | | | |
| ***Genotype*** | ***F13A1***  rs5985 | ***F13B***  rs6003 | ***FGA***  rs6050 | ***FGB***  rs1800790 | ***CFH***  rs1061170 | | | ***CRP***  rs876538 | | ***CRP***  rs2808635 | ***ABO***  rs657152 | | ***MTHFR***  rs1801133 | | **MTHFR**  rs1801131 | | | ***APOE***  rs7412/  rs429358 | ***TP53***  rs1042522 |
|  | *n (%)* | *n (%)* | *n (%)* | *n (%)* | *n (%)* | | | *n (%)* | | *n (%)* | *n (%)* | | *n (%)* | | *n (%)* | | | *n (%)* | *n (%)* |
| -- | 90 (73.2) | 103 (83.8) | 78 (63.4) | 81 (65.9) | 55 (44.7) | | | 74 (60.2) | | 62 (50.4) | 40 (32.4) | | 40 (32.5) | | 65 (52.8) | | | -- | 78 (63.4) |
| +- | 30 (24.4) | 18 (14.6) | 38 (30.9) | 39 (31.7) | 44 (35.8) | | | 39 (31.7) | | 51 (41.5) | 72 (58.5) | | 60 (488) | | 46 (37.4) | | | -- | 36 (29.3) |
| ++ | 3 (2.4) | 2 (1.6) | 7 (5.7) | 3 (2.4) | 24 (19.5) | | | 10 (8.1) | | 10 (8.1) | 11 (8.9) | | 23 (18.7) | | 12 (9.8) | | | -- | 9 (7.3) |
| *P* | 0.069 | 0.07 | 0.53 | 0.5 | 0.61 | | | 0.75 | | 0.28 | 0.08 | | **0.03** | | 0.56 | | | -- | 0.069 |
| - | 210 (85.4) | 224 (91.1) | 194 (78.9) | 201 (81.7) | 154 (62.6) | | | 187 (76.0) | | 175 (71.1) | 152 (61.8) | | 140 (56.9) | | 176 (71.5) | | | *E3:* 99 (80.5) | 192 (78.0) |
| *+* | 36 (14.6) | 22 (8.9) | 52 (21.1) | 45 (18.3) | 92 (37.4) | | | 59 (24.0) | | 71 (28.9) | 94 (38.2) | | 106 (43.1) | | 70 (28.5) | | | *E4:* 24 (19.5) | 54 (22.0) |
| *P* | **0.04** | **0.015** | 0.3 | 0.5 | 0.67 | | | 0.61 | | 0.56 | 0.74 | | **0.015** | | 0.81 | | | 0.47 | 0.05 |
| ***Genetic model*** | ORs (*P*) | ORs (*P*) | ORs (*P*) | ORs (*P*) | ORs (*P*) | | | ORs (*P*) | | ORs (*P*) | ORs (*P*) | | ORs (*P*) | | ORs (*P*) | | | ORs (*P*) | ORs (*P*) |
| (+-/++)/-- | 0.69  0.39-1.2  (0.20) | 0.54  0.28-1.04  (0.06) | 0.82  0.48-1.4  (0.48) | 0.78  0.44-1.37  (0.40) | 1.00  0.59-1.69  (0.98) | | | 1.06  0.62-1.81  (0.88) | | 1.04  0.62-1.74  (0.88) | 1.24  0.72-2.13  (0.44) | | **2.03**  **1.2-3.47**  **(0.009)** | | 0.84  0.5-1.41  (0.52) | | | -- | **0.54**  **0.32-0.92**  **(0.03)** |
| ++/(+-/--) | **0.24**  **0.065-0.9**  **(0.03)** | 0.23  0.04-1.1  (0.07) | 0.58  0.21-1.59  (0.29) | 0.64  0.14-2.9  (0.57) | 1.28  0.64-2.54  (0.47) | | | 1.48  0.52-4.24  (0.45) | | 0.54  0.23-1.26  (0.16) | 0.48  0.22-1.08  (0.07) | | 1.41  0.7-2.87  (0.34) | | 1.33  0.52-3.4  (0.54) | | | -- | 0.85  0.32-2.25  (0.9) |
| ++/-- | **0.23**  **0.06-0.88**  **(0.03)** | 0.22  0.04-1.08  (0.06) | 0.56  0.2-1.57  (0.27) | 0.70  0.15-3.24  (0.65) | 1.23  0.59-2.56  (0.57) | | | 1.48  0.51-4.31  (0.46) | | 0.59  0.24-1.42  (0.24) | 0.61  0.25-1.45  (0.26) | | 2.03  0.94-4.4  (0.07) | | 1.2  0.46-3.1  (0.71) | | | 1.28  0.65-2.54  (0.47) | 0.66  0.24-1.79  (0.56) |
| **VPI controls (n = 107)** | | | | | | | | | | | | | | | | | | | |
| ***Genotype*** | ***F13A1***  rs5985 | ***F13B***  rs6003 | ***FGA***  rs6050 | ***FGB***  rs1800790 | | ***CFH***  rs1061170 | ***CRP***  rs876538 | | ***CRP***  rs2808635 | | | ***ABO***  rs657152 | | ***MTHFR***  rs1801133 | | **MTHFR**  rs1801131 | ***APOE***  rs7412/  rs429358 | | ***TP53***  rs1042522 |
|  | *n (%)* | *n (%)* | *n (%)* | *n (%)* | | *n (%)* | *n (%)* | | *n (%)* | | | *n (%)* | | *n (%)* | | *n (%)* | *n (%)* | | *n (%)* |
| -- | 70 (65.4) | 79 (73.8) | 63 (58.9) | 76 (71.0) | | 48 (44.9) | 66 (61.7) | | 55 (51.4) | | | 40 (37.4) | | 53 (49.5) | | 52 (48.6) | -- | | 52 (48.6) |
| +- | 27 (25.2) | 21 (19.6) | 34 (31.8) | 27 (25.2) | | 42 (39.3) | 35 (32.7) | | 37 (34.6) | | | 49 (45.8) | | 39 (36.4) | | 47 (43.9) | -- | | 46 (43.0) |
| ++ | 10 (9.4) | 7 (6.6) | 10 (9.3) | 4 (3.7) | | 17 (15.9) | 6 (5.6) | | 15 (14.0) | | | 18 (16.8) | | 15 (14.0) | | 8 (7.5) | -- | | 9 (8.4) |
| - | 167 (78.0) | 179 (83.6) | 160 (74.8) | 179 (83.6) | | 138 (64.5) | 167 (78.0) | | 147 (68.7) | | | 129 (60.3) | | 145 (67.8) | | 151 (70.6) | *E3:* 90 (84.1) | | 150 (70.1) |
| + | 47 (22.0) | 35 (16.4) | 54 (25.2) | 35 (16.4) | | 76 (35.5) | 47 (22.0) | | 67 (31.3) | | | 85 (39.7) | | 69 (32.2) | | 63 (29.4) | *E4:* 17 (15.9) | | 64 (29.9) |

In bold significant ORs and P*-*values

| **Supplementary Table 2. Loadings of Principal Components (n=196)** | | | | | | | | |
| --- | --- | --- | --- | --- | --- | --- | --- | --- |
| **Variables** | **PC1** | **PC 2** | **PC 3** | **PC 4** | **PC 5** | **PC 6** | **PC 7** | **PC 8** |
| *F13A1 (rs5985)* | **-0.333** | 0.107 | 0.139 | 0.161 | **0.488** | **-0.321** | 0.193 | 0.241 |
| *F13B (rs6003)* | -0.024 | 0.140 | -0.248 | **0.480** | 0.211 | **0.555** | -0.127 | -0.006 |
| *FGA (rs6050* | 0.062 | 0.027 | 0.002 | **-0.561** | -0.135 | **0.490** | 0.069 | 0.058 |
| *FGB (rs1800790)* | -0.031 | 0.029 | 0.285 | **-0.300** | 0.110 | -0.087 | **-0.608** | -0.011 |
| *CFH (rs1061170)* | 0.207 | 0.248 | -0.084 | -0.123 | **0.306** | **-0.300** | **0.545** | -0.201 |
| *CRP (rs876538)* | 0.122 | **0.650** | **0.593** | 0.080 | -0.194 | 0.052 | 0.024 | -0.136 |
| *CRP (rs2808635)* | 0.200 | **0.767** | **0.447** | 0.135 | -0.092 | 0.046 | 0.017 | -0.072 |
| *ABO (rs657152)* | 0.175 | 0.105 | **-0.381** | -0.210 | -0.272 | 0.233 | **0.375** | **-0.326** |
| *MTHFR (rs1801133)* | 0.037 | **-0.500** | **0.506** | -0.154 | 0.255 | 0.181 | 0.237 | 0.060 |
| *MTHFR (rs1801131)* | -0.081 | 0.244 | **-0.493** | 0.051 | **-0.488** | **-0.378** | -0.076 | 0.262 |
| *APOE rs7412/rs429358* | 0.005 | 0.194 | -0.091 | **0.396** | 0.053 | **0.329** | 0.178 | **0.564** |
| *TP53 (rs1042522)* | -0.045 | 0.120 | **-0.356** | **0.317** | **0.377** | 0.029 | -0.228 | **-0.552** |
| Age | 0.207 | **-0.441** | 0.272 | **0.444** | **-0.312** | 0.046 | 0.066 | -0.206 |
| Methylation | **-0.345** | **0.381** | -0.263 | **-0.457** | **0.314** | 0.169 | -0.079 | 0.083 |
| IL10 | **0.912** | -0.032 | 0.009 | -0.096 | 0.056 | -0.009 | -0.023 | 0.025 |
| IL17A | **0.815** | 0.012 | -0.160 | 0.043 | 0.098 | -0.074 | -0.054 | 0.090 |
| IL23 | **0.915** | 0.031 | -0.047 | -0.057 | 0.115 | -0.059 | -0.066 | 0.061 |
| IL6 | **0.921** | -0.008 | -0.091 | -0.008 | 0.120 | 0.014 | -0.096 | 0.126 |

In bold the main loadings with cut-off > 0.30

| **Supplementary Table 3. Principal Component regression analysis (n=196)** | | | |
| --- | --- | --- | --- |
| **PCs** | **OR (95%CI)** | ***P*** | **Main Loadings of PCs** |
| PC1 | 1.895 (1.358-2.644) | **0.000** | *F13A1,* Methylation, CKs |
| PC2 | 0.552 (0.404-0.754) | **0.000** | *CRP, MTHFR(1),* Age, Methylation |
| PC3 | 1.482 (1.103-1.992) | **0.009** | *CRP, ABO, MTHFR, TP53* |
| PC4 | 1.449 (1.073-1.955) | **0.015** | *F13B, FGA, FGB, APOE, TP53,* Age, Methylation |
| PC5 | 0.609 (0.448-0.825) | **0.002** | *F13A1, CFH, MTHFR(2), TP53,* Age, Methylation |
| PC6 | 0.829 (0.623-1.103) | 0.198 | *F13A, F13B, FGA, CFH, MTHFR(2), APOE* |
| PC7 | 1.071 (0.808-1.420) | 0.632 | *FGB, CFH, ABO* |
| PC8 | 1.044 (0.788-1.385) | 0.763 | *ABO, APOE, TP53* |

In bold significant ORs and *P-*values
